# Hind-foot correction and stabilization by pins in plaster after surgical release of talipes equino varus feet in older children

**DOI:** 10.1186/1749-799X-5-42

**Published:** 2010-07-02

**Authors:** Mohamed M El-Sayed, Osama A Seleem

**Affiliations:** 1 Mohamed M El-Sayed, Consultant & Lecturer of Pediatric Orthopedic Surgery, Department of Orthopedics & Traumatology, Tanta University, 3111, Tanta, Gharbia, Egypt; 2 Osama A Seleem, Consultant & Assistant Professor of Orthopedic Surgery, Department of Orthopedics & Traumatology, Tanta University, 3111, Tanta, Gharbia, Egypt

## Abstract

Congenital talipes equino varus (CTEV) is a three dimensional deformity and is one of the most common congenital abnormalities affecting the lower limb and can be challenging to manage. Hind-foot deformity is considered the most difficult to treat. Unfortunately, the calcaneus is often small and thus difficult to control during casting after surgical release in severe or relapsed cases. We used three pins to control and maintain the hind foot correction, after surgical release, during casting in 47 cases (59 feet). We introduced a modified, coronal plane, transverse calcaneal pin. This pin is inserted from medial to lateral through the calcaneus to correct the varus mal-positioning of the calcaneus in the sagittal plane and to provide a better control on the small sized, hind-foot during casting. We paid special attention to the final hind-foot deformity after surgery, and the results were favorable after the application of this transverse pin.

## Introduction

CTEV is a complex deformity that has a tendency to recur until the age of six or seven years [1]. Recently after the introduction of the Ponseti method, there is almost a universal agreement on the non-operative management of CTEV [2-6].

It is likely that a small number of clubfeet will require surgery even after expertly applied non-operative treatment. In some patients, either failure to obtain a complete correction or failure to maintain the correction occurs [6].

In those patients with severe relapsed deformities, the calcaneus is often small and difficult to control during casting. A residual varus mal-positioning of the hind-foot may occur, after complete adequate surgical release. We used three pins to control and maintain the hind foot correction in the normal position (about 5° valgus) during casting in the studied 59 feet.

## Patients and Methods

Between Oct. 2003 and Sept. 2009, 47 cases (59 feet) of CTEV, were operated upon using the below described surgical technique. The parents gave the informed consent to include their kids into the study. There were 35 unilateral cases and 12 bilateral cases. The duration of previous conservative management ranged from 5 to 22 months, with a mean of 12 months.

In all cases, a trial of conservative management, using the Ponseti method, was strongly suggested at our center which was not accepted by the parents of all the children included in this study. History of previous treatment is summarized in (Table 1) and demonstrates a history of good initial results and deformity correction following previous conservative management that was reported In 16 unilateral, and 5 bilateral patients using the Ponseti method. This was not maintained and the deformity recurred in this group of patients, and that was attributed to the poor family compliance, inadequate orthosis, and/or follow-up. History of previous surgical intervention was reported in 19 unilateral and 7 bilateral cases (33 feet), and the deformity recurred despite reported initial post-operative adequate reduction by the parents.

**Table 1:** History of previous treatment

	Unilateral patients	Bilateral patients	Number of feet
Non-surgical treatment	16	5	26

Surgical treatment	19	7	33

Total	35	12	59

**Figure 1:**
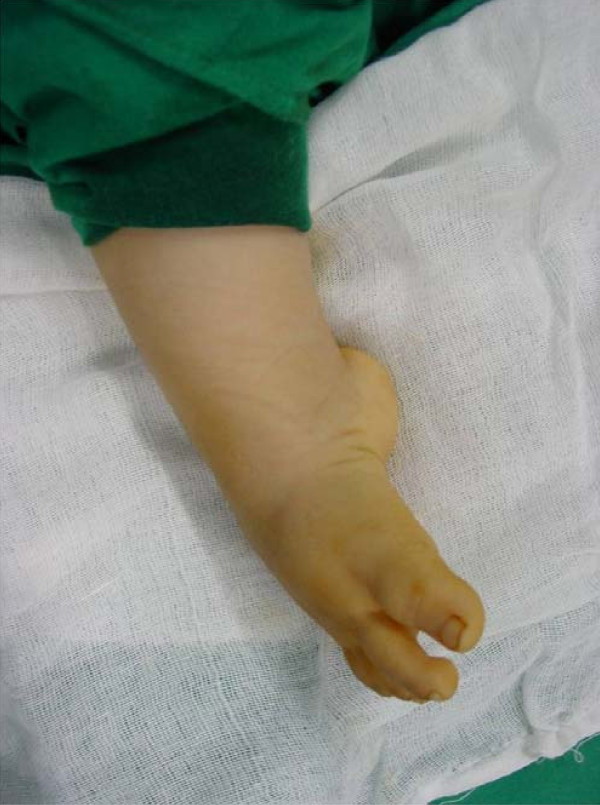
**Preoperative photo (with patient under general anesthesia), Rt. very severe resistant CTEV in a 22 months male pt., after 12 months of serial casting at another center**. Notice the medial and posterior deep skin creases, and the severe equinus deformity of the foot.

The age of the patients at the time of surgery ranged from 18 to 59 months, (mean of 29 months). Based on the Diméglio classification [7], the deformity was very severe in 51 feet, and severe in 8 before surgery. (Table 2, Figure 1).

**Table 2:** Severity of foot deformity before surgery according to Diméglio classification, [7].

Classification grade	Type	Score	Number of feet	Frequency (%)
benign	I	<5 points	0	0%

Moderate	II	= 5 - <10	0	0%

Severe	III	= 10- <15	8	13.5%

Very severe	IV	= 15- <20	51	86.5%

**Figure 2:**
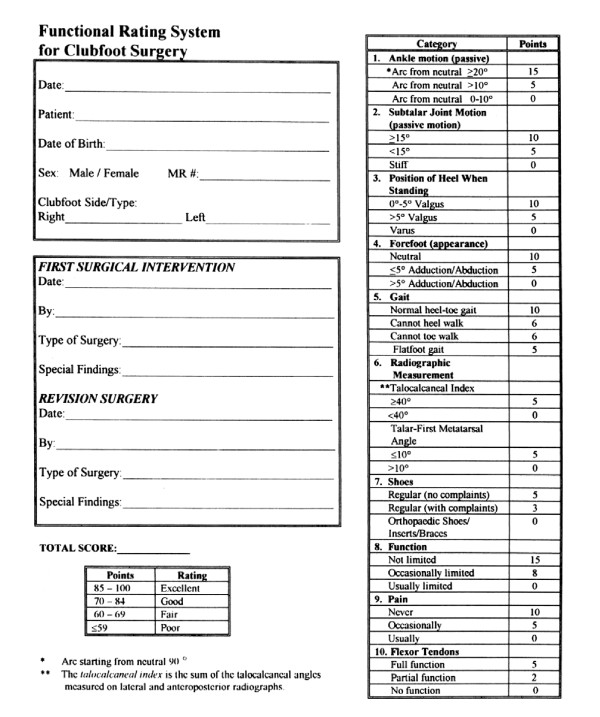
**The functional rating system **[8], **for clubfoot revision surgery**.

The functional rating system reported by Cummings, et al, [8] was used for evaluation of previously surgically treated 26 patients (33 feet). This rating system was developed to determine the need for revision surgery in relapsed or recurrent deformity. Scores of <60 points (total 100) indicate the need for revision according to the authors (Figure 2). All the evaluated 26 cases had had a poor (<60 points) functional score before the index surgery, the range was from 42 to 59 with a mean of 51 points.

### The surgical technique

The Turco [9], oblique or hockey-stick posteromedial incision was used in 19 feet, while the Cincinnati [10], incision and approach was used in 40 feet, (the same approach was used in revision cases including 19 Turco incisions and 14 Cincinnati incisions, while the Turco approach was used in only 5 cases and the Cinncinati approach was used in 21 previously conservatively treated feet). After a complete thorough surgical release was performed, the talus was inwardly rotated, and the navicular was reduced on the head of the talus. When the navicular was properly reduced, the medial tuberosity should have been prominent. If it was flush with the medial aspect of the talar head and neck, this means it was over-reduced laterally. It should, however, be flush with the dorsum of the talar head.

The "à la carte" approach to the clubfoot as described by Bensahel et al.[11], i.e., do only what is necessary to get a good correction of the foot, was used to achieve full correction of the deformity present with the least soft tissue dissection possible. But complete adequate release was obtained and ensured in all the cases.

#### Reduction and Fixation

After adequate surgical release and deformity correction, a modified three pins technique was used to maintain the foot in the corrected position. The pins used were 1.2 -1.4 mm smooth Kirschner wires (KW), according to the age of the patient and the size of the affected foot. The three pins were used as follows;

a. The first pin (Talonavicular wire); Simons [12], reported that this pin should be placed centrally in the head of the talus and drilled in a retrograde fashion until it emerges at the posterolateral ridge of the talus, while Carroll [13], passed this wire from the posterolateral corner of the talus longitudinally toward the talar head. The navicular was then reduced, and the pin was driven across the joint. The Carroll method of reduction and fixation of the talo-navicular joint was used in all the studied cases in this study. In the sagittal plane, the pin should be in line with the first metatarsal. This pin was used as a joystick to rotate the talar body internally while the navicular was pushed into abduction and onto the true talar head. At this point, we made sure that the reduction was anatomic and that no rotation of the navicular has occurred as a result of pivoting on lateral soft tissue or calcaneal obstruction.

b. The second pin (modified coronal wire); which is the additional wire used in this study, was inserted into the calcaneus in the coronal plane. This wire was inserted from medial to lateral direction, about 1-1.5 cm anterior to the posterior end of the calcaneal tuberosity. At this point the pin was inserted under vision to avoid injury of the calcaneal branch of the posterior tibial nerve. The calcaneus needs to be rotated so that the tuberosity moves medially away from the fibula. In this position, the cuboid was reduced on the end of the calcaneus. Pinning of the calcaneo-cuboid was not used in this study. This coronal calcaneal pin allowed for proper positioning of the calcaneus into the normal 5° valgus, provided better correction of the equinus deformity of the calcaneus, and enabled a better hand grip and control of the hind foot during casting after surgery.

c. The third pin (subtalar wire); After complete subtalar release, and correction of the hind-foot varus and control of the calcaneus to ensure its normal positioning in the sagittal plane, the subtalar joint was fixed. This pin was introduced through the plantar surface of the calcaneus, across the subtalar joint and into the talus. It should not pass into the ankle joint. Care was taken to ensure that the calcaneus was not tipped into varus or valgus, and this was guaranteed by the proper positioning and control of the second coronal wire.

#### Intraoperative Assessment

**Figure 3:**
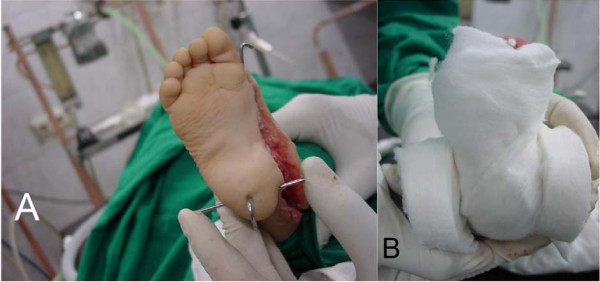
**Intraoperative photos showing A; complete deformity correction with a straight lateral border of the foot, and the 3 pins inserted into position to provide better control during casting, and B; the circular "orthopad" pieces in position to cover the ends of the second coronal pin medially and laterally**.

Once the reduction and pinning have been completed, the position of the foot was then rechecked with the knee in 90° of flexion. It must be plantigrade without a varus, valgus, supination, or pronation deformities. (Figure 3-A). The thigh-foot axis should be outwardly rotated 0° to 20°.

The Achilles tendon was repaired with the ankle in 10° of plantar flexion so that there was some tension on it when the foot was in the neutral position. The wound was then closed. A special padding for the transverse wire was used to provide a better hand grip during casting. About 2 cm wide large circles of orthopad were placed, to cover the prominent ends of the transverse wire in the coronal plane (Figure 3-B). The hind-foot position was maintained holding the orthopad into the desired valgus position. Immobilization by an above-the-knee cast was applied.

#### Postoperative Management

A caudal block at the end of the procedure was used. If the cast was applied at an under corrected position (specially equinus) to properly close the wound, one week postoperatively, the cast was changed with the foot plantigrade and outwardly rotated and the knee flexed 90°. The cast was worn for four to six weeks, after which the pins were removed, and weight-bearing was allowed about six to eight weeks post-operatively.

**Figure 4:**
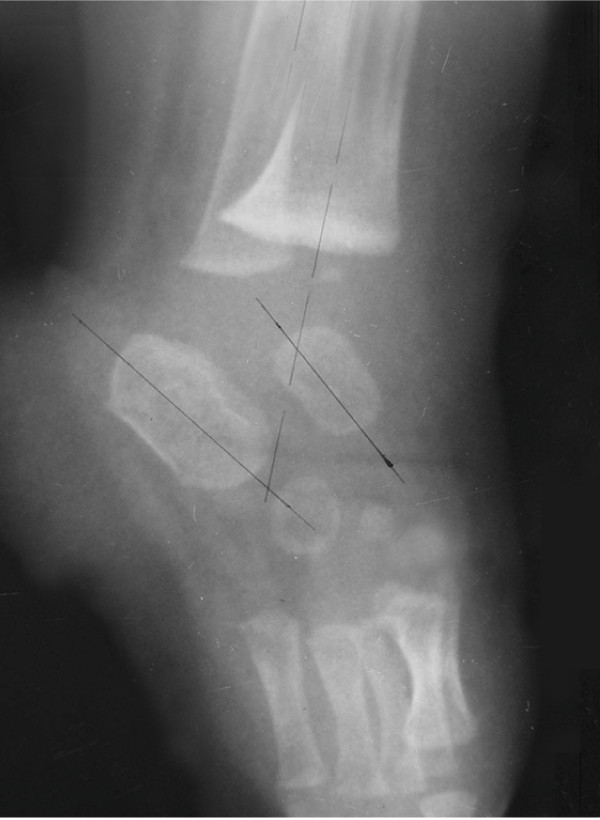
**Preoperative plain lateral radiograph of the Rt. foot, with passive dorsiflexion of the foot, showing parallelism of the talus and calcaneus, severe equinus of the calcaneus and an acute tibio-calcaneal angle**.

**Figure 5:**
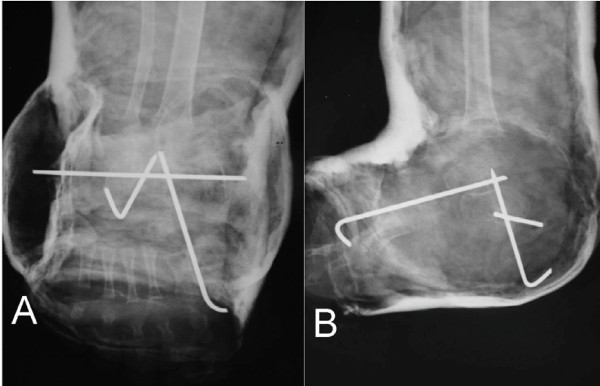
**A; AP post-operative X-ray, showing convergence between the talus and calcaneus (33° Kites angle), and AP positive(5°) talus-1**^**st **^**MT angle, B; lateral view showing immediate correction of the lateral talo-calcaneal angle (36°), and an obtuse tibio-calcaneal angle**.

The operative time ranged from 45 to 95 minutes (mean of 55). Standard radiographic examination was performed preoperatively in older children (Figure 4), immediately postoperatively (Figure 5), after removal of the wires, and at the final follow-up period. The antero-posterior (AP) talo-calcaneal angle, (Kites angle), the AP talus -first metatarsal angle, the lateral tibio-calcaneal angle, and the lateral talo-calcaneal angle were measured.

The follow-up period ranged from 18 to 67 months with a mean of 32 months.

A modified classification was used after measurement of the hind foot axis using a goniometer to measure the angle between the long axis of the leg and the calcaneus (heel position during standing). This was used to evaluate the final position of the hind foot at the final follow up visit.

## Results

The preoperative AP Kites angle ranged from 5°-16°, with a mean of 9°, while the AP talus-1^st ^MT angle was always negative in preoperative films, with a range of -30° to -65°, and a mean of -43°. In the lateral view, the preoperative talo-calcaneal angle ranged from 0°-14° (parallelism of the talus and calcaneus), with a mean of 5°. The lateral tibio-calcaneal angle was always an acute angle with values from 45° to 80°, and a mean on 55°.

The postoperative radiographic measurements at the final follow-up visit, were as follows; the AP Kites angle ranged from 20°-36°, with a mean of 28°. The AP talus-1^st ^MT measured 0°-14°, with a mean of 9°. The lateral talo-calcaneal angle was between 31° to 42°, with a mean of 36°. The lateral tibio-calcaneal angle was corrected to an obtuse angle, with values from 103° - 135°, and a mean of 115°, (Table 3).

**Table 3:** Preoperative and postoperative radiographic evaluation.

	Preoperative values	Postoperative values
AP Kites angle range:	5°-16°	20°-36°
Mean:	9°	28°

AP Talus-1^st ^MT range:	-30° to -65°	0°-14°
Mean:	-43°	9°

Lat. Kite angle range:	0°-14°	31° to 42°
Mean:	5°	36°

Lat.tibio-calcaneal range:	45° to 80°	103° - 135°
Mean:	55°	115°

The clinical hind-foot axis measurement at the final follow-up visit revealed values from 0° to 11° valgus with a mean of 5°.(Table 4).

**Table 4:** Evaluation of the final heel position during standing using a modified rating system.

Standing hind-foot angle	Number of feet	Percent
6-10° valgus	12	20.4%

1- 1- 5° valgus	44	74.6%

≤ 0° varus	3	5.0%

## Complications

1. Seven feet developed wound dehiscence after removal of sutures two weeks after surgery, (they were operated upon using the Cincinnati approach). All the feet were maintained in the corrected position and granulation tissue took place. This complication did not affect the final clinical outcome.

2. Superficial wound infection took place in 8 feet and they were treated adequately with proper antibiotics and sterile dressing of the wound. All the wounds healed and left no unfavorable results.

3. Removal of the talo-navicular wire was reported in one patient 3 weeks after surgery. The pin was brought into the clinic by the parents, (they stated that it was loose and they noticed that the on-top dressing and the wire were removed by their child).

**Figure 6:**
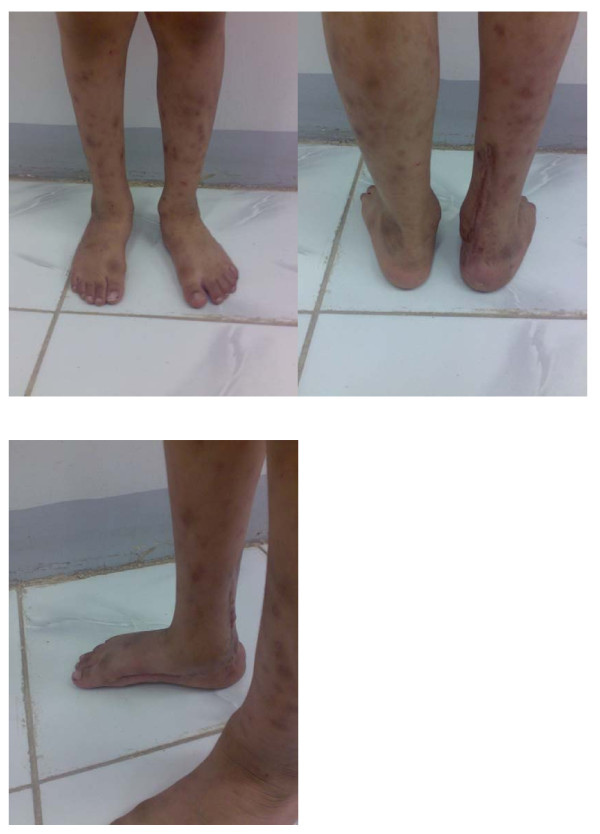
**Final clinical presentation of the patient 38 months after surgery showing an excellent clinical outcome, with a comparable valgus hind foot angle of the right foot**.

Clinical examination at the final follow-up revealed that all the patients had a pain-free, plantigrade, and mobile feet with normally positioned (although looks smaller comparative to the healthy side in unilateral cases), hind feet (Figure 6). Hind-foot movements were examined and showed within normal (5°-10°) inversion values and eversion values (10°-20°), at the subtalar joint.

The functional rating system described above was used at the final follow-up visit to re-evaluate the feet. All the cases had excellent, (≥ 85 points), scores. The scores ranged between 85 and 95 points with a mean of 88 points.

## Discussion

CTEV is a three-dimensional deformity that must be understood before attempting corrective measures. Medial and plantar displacement of the navicular, cuboid, and calcaneus around the talus result in an inverted or varus hind-foot, and the entire complex rests in quines [14].

Nowadays, although there is almost a universal agreement on non-surgical management of CTEV [15-19], and also reports of trials of application of the Ponseti technique in severe arthrogrypotic club feet [20], there are still reports of early recurrence of the deformity, and it is likely that a small number of clubfeet will require surgery even after expertly applied non-operative treatment [21].

Along with other complications of poor parents compliance, long duration of casting, incomplete correction of the deformity, recurrence of the deformity, difficulty of treatment of old neglected cases with severe deformity and finally parents refusing proposed non-operative trials, surgical treatment will be the indicated line of treatment in few relapsed severely deformed feet.

The parents of all the patients included in this study refused the non-operative technique, although it was strongly recommended, even in severe relapsed cases after failure of previous surgical management, especially in the younger age group.

Transfixion of the talonavicular joint with a fine Kirschner wire ensures that this correction will be maintained [12]. Some of the failures after previous soft-tissue surgery resulted from a loss of the initial correction when only a plaster cast was used to stabilize the reduction [9].

Here it is of value to mention that, although the calcaneus is not as deformed as the talus, displaying only slight shortening and widening with mild medial bowing. It is integral to the positional deformities of CTEV: quines, varus, and adduction [22].

We believe that the equino-varus deformity of the calcaneus is the most difficult to correct in relapsed severe cases. In infants under three months of age, manipulative treatment by conventional methods is usually successful; but in infants over four months of age, it may not be possible by manipulative treatment to get the calcaneus into the exact position desired, even when lengthening of the Achilles tendon is performed. Recurrent deformities, the so-called rocker-bottom deformities, caused by poor treatment, and untreated deformities in older children are particularly difficult to treat by manipulative methods. This was also approved by many authors and various techniques have been suggested for the treatment of these more complicated deformities [23-25].

It was also noted that residual hind-foot varus and/or cavus deformities of the heel were among the most common complications after surgical treatment of CTEV, even after the use of the traditional (talonavicular, and subtalar wires), pins for stabilization of the corrected feet [26].

We paid special attention to the hind-foot deformity, and introduced a transverse (coronal plane) wire into the calcaneus to use it as a joystick to control the adequately released bone into the coronal plane to precisely correct the supination and varus deformities into the normal desired position. This wire also provided better correction of the quines deformity of the calcaneus, which was proved by the immediate improvement of the lateral talo-calcaneal and the lateral tibio-calcaneal angles. Finally this wire was of great help during casting after closure of the wound as it allowed better handling and grip of the small slippery heel within the cast.

Early clinical and radiological assessment of all the cases at periodic intervals showed comparable favorable results in accordance with other studies using the Ponseti method [27,28], as well as, after surgical soft tissue release [29-31]. In addition we paid special attention to the hind-foot axis at the final follow-up and modified a classification system for our patients based on the clinical angle measured using a goniometer (Table 4). There was a favorable hind foot positioning in about 95% of the studied cases at the final follow-up visit. Only 3 feet ended with a 0° hind-foot axis and was considered as a varus heel, and non-favorable result. All those complicated cases with residual final varus deformity presented with very severe deformity, had had previous surgical intervention, and were older than 36 month.

This modified suggested pinning technique provided better control and correction of the hind-foot deformity. During casting this was of particular importance as it enabled the surgeon to have a good grip on the small sized calcaneus in all planes possible. By the end of the follow-up period, all the patients showed excellent functional rating scores. We think that we should follow the cases for longer durations to provide a long-term results of this technique, but we believe that our early clinical and radiographic values are promising to manage this severe recurrent deformity when surgical intervention is considered in very severe CTEV cases.

## Competing interests

The authors declare that they have no competing interests.

## Authors' contributions

Both authors shred equally in performing, writing, editing and revising this study. Plus, all the authors read and approved this final manuscript.
